# Factors affecting primary care physician decision-making for patients with complex multimorbidity: a qualitative interview study

**DOI:** 10.1186/s12875-022-01633-x

**Published:** 2022-02-05

**Authors:** Linnaea Schuttner, Stacey Hockett Sherlock, Carol Simons, James D. Ralston, Ann-Marie Rosland, Karin Nelson, Jennifer R. Lee, George Sayre

**Affiliations:** 1 Health Services Research & Development, VA Puget Sound Health Care System, 1660 S Columbian Way, Seattle, Washington, 98108 USA; 2 Department of Medicine, University of Washington, Seattle, WA USA; 3 Comprehensive Access & Delivery Research and Evaluation (CADRE) Center, VA Iowa City Health Care System, Iowa City, IA USA; 4 Carver College of Medicine, University of Iowa, Iowa City, IA USA; 5 Kaiser Permanente Washington Health Research Institute, Seattle, WA USA; 6 Department of Health Systems and Population Health, University of Washington School of Public Health, Seattle, WA USA; 7 VA Center for Health Equity Research and Promotion, VA Pittsburgh Healthcare System, Pittsburgh, PA USA; 8 Department of Medicine, University of Pittsburgh, Pittsburgh, PA USA; 9 Department of Urology, University of Washington, Seattle, WA USA

**Keywords:** Multimorbidity, Veterans, Primary care, Qualitative research, Clinical decision-making

## Abstract

**Background:**

Patients with multiple chronic conditions (multimorbidity) and additional psychosocial complexity are at higher risk of adverse outcomes. Establishing treatment or care plans for these patients must account for their disease interactions, finite self-management abilities, and even conflicting treatment recommendations from clinical practice guidelines. Despite existing insight into how primary care physicians (PCPs) approach care decisions for their patients in general, less is known about how PCPs make care planning decisions for more complex populations particularly within a medical home setting. We therefore sought to describe factors affecting physician decision-making when care planning for complex patients with multimorbidity within the team-based, patient-centered medical home setting in the integrated healthcare system of the U.S. Department of Veterans Affairs, the Veterans Health Administration (VHA).

**Methods:**

This was a qualitative study involving semi-structured telephone interviews with PCPs working > 40% time in VHA clinics. Interviews were conducted from April to July, 2020. Content was analyzed with deductive and inductive thematic analysis.

**Results:**

23 physicians participated in interviews; most were MDs (*n* = 21) and worked in hospital-affiliated clinics (*n* = 14) across all regions of the VHA’s national clinic network. We found internal, external, and relationship-based factors, with developed subthemes describing factors affecting decision-making for complex patients with multimorbidity. Physicians described tailoring decisions to individual patients; making decisions in keeping with an underlying internal style or habit; working towards an overarching goal for care; considering impacts from patient access and resources on care plans; deciding within boundaries provided by organizational structures; collaborating on care plans with their care team; and impacts on decisions from their own emotions and relationship with patient.

**Conclusions:**

PCPs described internal, external, and relationship-based factors that affected their care planning for high-risk and complex patients with multimorbidity in the VHA. Findings offer useful strategies employed by physicians to effectively conduct care planning for complex patients in a medical home setting, such as delegation of follow-up within multidisciplinary care teams, optimizing visit time vs frequency, and deliberate investment in patient-centered relationship building to gain buy-in to care plans.

**Supplementary Information:**

The online version contains supplementary material available at 10.1186/s12875-022-01633-x.

## Background

Patients with multiple chronic conditions are growing in prevalence [1] and primary care physicians (PCPs) expend significant effort and time in caring for these patients [2, 3]. These patients are at greater risk for adverse outcomes, arising from illness burden, polypharmacy, or care fragmentation [4–8]. Physician decision-making is a critical part of patient-centered, evidence-based care planning for these patients, that is, developing personalized support plans for patients tailored to their individual needs [9]. However, for patients with multimorbidity and increased psychosocial complexity, [10] PCPs must often make care decisions in the absence of clear clinical practice guidelines or with a minimum of relevant clinical evidence for support [5, 11, 12]. Providing high-quality, patient-centered care requires individualizing care plans for these patients that account for their health conditions, psychosocial context, and preferences.

Conceptual frameworks examining clinician decision-making have been previously described [13, 14]. The clinical decision-making process is dynamic, entailing understanding and prioritizing a patient’s care concerns, deciding on diagnostic testing, interpreting medical information, recommending a care plan, and getting feedback on treatment response. While general aspects of decision-making govern most medical encounters, complex patients with multimorbidity have unique aspects to consider. For example, decisions for these patients must account for disease interactions, conflicting disease recommendations, finite patient capacity for self-management, and impacts from treatment burden [10, 15, 16].

Improving clinical decision-making in the pursuit of quality and patient-centeredness requires a clear understanding of relevant factors affecting this process. Effort has gone into understanding this topic for a broader patient population [17–20]., but only a few studies have been specific for clinical decision-making for complex or multimorbid populations [7, 21]. Additionally, to our knowledge, none have occurred with U.S. PCPs caring for patients exclusively within patient-centered medical homes. The healthcare system of the U.S. Department of Veterans Affairs, the Veterans Health Administration (VHA) is an integrated health system with over 900 primary care clinics serving almost 9 million veterans. Primary care is delivered within a patient-centered medical home model consisting of a primary care provider, nurse care manager, clinical associate, and administrative assistant, with affiliated interdisciplinary support members including mental health, pharmacy, and social work. This medical home model may have additional influences on primary care clinical decision-making for PCPs. We sought to clarify this evidence gap by conducting qualitative interviews to understand factors affecting PCP care planning decisions for patients with multimorbidity and increased psychosocial complexity within the integrated VHA system.

## Methods

### Study overview and setting

Physicians participated in one-on-one, semi-structured telephone interviews from April to July 2020. Data were analyzed using deductive and inductive thematic analysis conducted using a pragmatic framework and influenced by a phenomenological perspective, given the objective to understand PCP experience and derive generalizable, actionable factors affecting clinical decision-making [22]. We followed the standards for reporting qualitative research (SRQR) guidelines (Additional File 1) [23]. This work was designated as non-research, quality improvement for the VHA Office of Primary Care in accordance with the national VHA Office of Research and Development policy of the U.S. Department of Veterans Affairs. This exempts the work from further VHA Institutional Review Board (IRB) review. Documentation of this process can be provided upon reasonable request. Verbal informed consent to participate and be recorded was obtained from all participants.

### Researcher characteristics and reflexivity

This study was led by L.S., a practicing PCP in the VHA and health services researcher. L.S. worked with C.S., a Bachelors’ level research associate with expertise in interviewing clinicians, and G.S., a highly experienced qualitative research scientist with expertise in operational-research partnerships, phenomenology, and pragmatic qualitative methodology, to create the interview guide. Interviews were conducted by C.S. with supervision and review by L.S. Data analysis and interpretation was conducted by L.S. alongside S.H.S., a Masters’ level qualitative research analyst who has experience working on operationally-partnered, clinically oriented projects within the VHA. Assistance in thematic interpretation and conceptual alignment came from three PCPs and health services researchers: J.D.R. (a non-VHA PCP with specialization in clinical decision-support and patients with multimorbidity), A.M.R. (a VHA PCP, with specialization in complex and high-risk populations), and K.N. (also a VHA PCP, with specialization in primary care delivery models). J.R.L., a Masters’ level, non-VHA qualitative research analyst, and G.S. provided methodology advice to L.S. and S.H.S. during analysis. None of the authors had personal relationships with the participants.

### Participants

We conducted interviews with primary care physicians within the VHA (MD, DO, or equivalent physician license). We purposively sampled attending physicians working clinically at least 40% (2 days a week) to capture perspectives from those providing a substantial component of direct primary care (versus administrative or research duties). As hospital-affiliated and community-based clinics provide different environments, [24] we stratified sampling 1:1 between those in VHA medical center-affiliated vs. community-based clinics. Participant email addresses and demographics were from VHA administrative data [25]. A total of 475 physicians were sent recruitment emails; 25 responded. Initial emails were from L.S. and specified that input was being sought on how PCPs approach care decisions for patients with multimorbidity.

### Interviews and data collection

C.S. conducted semi-structured telephone interviews. Audio-recorded interviews were approximately 20–30 min long and transcribed verbatim by project staff. The 12-question interview guide (Additional File 2) asked physicians to think about decision-making from a recent encounter for a complex patient with multiple chronic conditions (defined by the interviewer on request as two or more chronic diseases for either physical or mental health diagnoses; complexity was self-defined according to the physician). Probes and additional open-ended questions were used by C.S. as needed to elicit participant experiences. Questions were based on pre-defined conceptual models of complex multimorbidity and delivery of patient-centered care [10, 26]. C.S. and L.S. met weekly to discuss fidelity to the project objective and review within-interview accuracy checks (paraphrasing, interpretive statements). After reaching the anticipated minimum of 20 interviews, [27] C.S. and L.S. reviewed every 2–3 interviews for thematic saturation [28]. Twenty-five interviews were conducted, two were discarded prior to analysis due to unsalvageable audio. Thematic saturation was reached after 23 interviews were analyzed.

### Data analysis

Transcripts were analyzed using a deductive and inductive thematic approach, primarily from a pragmatic framework influenced by a phenomenological perspective to concentrate on actionable, operationally-relevant findings [22, 29]. S.H.S., using de-identified transcripts, first coded all segments related to factors and pulled relevant segments into a summary template format. This initial deductive review allowed for a more rapid timeline for analysis, concordant with the operationally-partnered nature of this research. Deductive factors were defined as influences perceived to shape clinical decision-making experiences by the participant. These segments were then reviewed by L.S. and S.H.S. independently to develop inductive codes. The team met to reach consensus and develop a final codebook. A final transcript review for additional codes was conducted by S.H.S. Visual mapping of interactions was reviewed to ensure minimal conceptual overlap between codes. After finalizing the codebook, inductive thematic analysis was conducted independently by L.S. and S.H.S. reaching consensus for final themes. MAXQDA software [30] was used for coding and data management, with additional processing (e.g., codebook development) using Microsoft Excel.

## Results

Of 23 participating physicians, most were MDs (*n* = 21) in hospital-affiliated clinics (*n* = 14). Interviews were conducted with physicians across all regions of the VHA. Demographics of the participants are in Table 1. Developed themes for factors affecting decision-making for complex patients with multimorbidity are described below; Tables 2, 3, and 4 provides additional representative quotes.

**Table 1:** Demographics of participating physicians completing qualitative interviews

Demographics	No. (***N*** = 23)	%
Female sex	14	61
Non-MD degree (DO, MBBS)	2	9
Clinic location		
VA medical center affiliated	14	61
Community affiliated	7	30
Other	2	9
Region		
Midwest	7	30
Southeast	7	30
West	5	22
Northeast	2	9
Southwest	2	9
Medical practice type		
General primary care	17	74
Women’s clinic	4	17
Homeless care clinic	1	4
Home-based primary care	1	4
Time devoted to clinical role, Mean (SD)	78% (21%)	–
Years in practice after residency, Mean (SD)	20.5 (11.3)	–

**Table 2:** Representative Quotes of Internal Factors

***Internal factors of individuals affect care planning decisions***	
***Subtheme #1. Decisions are tailored to individual patients.***	
Severity of concern/health	“I first prioritize what the veteran’s goal of the visit is, but I also look at what would be most threatening, in terms of their long-term health. If the issue at that time is that the COPD or asthma is uncontrolled, and they’re wheezing and short of breath, I’d be more likely to address that.” (P00)
Response to observable data	“You prioritize with your vitals – if […] his blood pressure is extremely high, got to really address that; if his sugars are really extremely high. I actually usually do address both of those.” (P13)
Decisions are unique	“For some people their struggle is […] clinical. For other people their struggling is social. For other people it’s economic. For other people it’s mental health.” (P17)
***Subtheme #2. An underlying PCP style or habit guides decisions.***	
Physicians have a style or preferred approach	“Part of my job is to be the coach and encourager and know that this is a lifelong process. You’ve got to make small changes that are permanent, but you can’t try and make everything change all at once.” (P11)
Documentation is bidirectional with care decisions	“If you have time to prep, that’s always a good thing, because you can either go over the home monitoring stuff or like I said, go back to your previous notes: 'OK, I know I needed to ask about this, because I made a note about it in my last note.'” (P06)
***Subtheme #3. Care planning occurs towards an overarching goal for care.***	
Stability or status-quo	“I ask him: ‘Are you status-quo today or is there something different going on?’ And then I look to these others [to] make sure that’s stable.” (P21)
Patient goals, acute needs take priority	“Whatever the patient feels to be the absolute necessary to address, but there are times we start examining them and other things take over because they absolutely need to be addressed. Then anything else like chronic disease that needs to be addressed.” (P01)

**Table 3:** Representative Quotes of External Factors

***External factors within the environment or context influence care planning decisions***	
***Subtheme #4. Patient access to resources constrains care plans****.*	
Time tradeoffs are inherent	“Either you address the main concern […] and have them come back later. Or, if you can address everything in one visit, then you’re bringing [them] back less times.” (P04)
Resource availability	“Some of these patients, they come from really far away and we don’t have an MRI and CT where we’re at, we’re like a peripheral site. I wanted some imaging of his back, [but] it means driving down even further.” (P15)
***Subtheme #5. Organizational structures provide boundaries.***	
Tasks pair to visit modality	“We need to be able to maybe see the complex ones more often so that we can uncomplex them. Once they’re stabilized, twice a year is fine, but a lot of these, you’ve got to be seen a lot more often – and a lot of it needs to be face to face; it’s not going to work by phone.” (P23)
Organizational peculiarities	“Sometimes patients get mixed messages, they get a different message from me and a different message from [...] their civilian provider.” (P19)

**Table 4:** Representative Quotes of Relationship-Based Factors

***Relationship-based factors across individuals affect care planning decisions***	
***Subtheme #6. Care planning is collaborative for complex patients.***	
Team collaborates on workload and provides collateral information	“You have to feel it out at first, just be cautious, and as the clinical situation develops, then you just get a good sense of what’s going on. That’s what I did in that scenario, just bring in multiple players and [...] you can see them in the home, the social workers getting the social aspect of it, and then you just really go with your gut feeling.” (P03)
Primary care has a defined scope	“Specialty care needs to take care of the consults. Stop putting patients on primary care all the time and follow up on that.” (P04)
***Subtheme #7. Decisions are affected by dynamic patient relationships.***	
Advance trust and buy-in	“She wanted to stop smoking, lose weight, use CPAP because she had sleep apnea, and eat better. Those are big, and so I had to get her to see how her current lifestyle was preventing her from being able to do that, so therefore I got more buy-in.” (P08)
Physician internal state	“I explained that that was my reason for not saying that he was legally blind, but he wanted me to change the form anyways [...]. I don’t know if that affected care; it affected me as a provider – I felt like it was another layer of drama and frustration in trying to provide care for him.” (P09)

Factors affecting PCP decision-making during care planning were developed within three main themes: internal influences related to the individual patient or PCP; external influences from the context or environment; and relationship-based factors that extended across individuals. Internal factor subthemes included the tailoring of decisions to specific individual patients, the underlying style or a fixed habit of the PCP guiding decisions, and care planning decisions occurring in response to an overarching goal for care. External factor subthemes included patient access to resources, and the boundaries established by organizational structures. Relationship-based subthemes included the importance of collaborative care, and the dynamic relationship between PCPs and patients that affected care planning decisions.

### Internal factors related to the individual patient or PCP affect care planning decisions

#### Subtheme #1. Decisions are tailored to individual patients

Physicians considered unique patient characteristics and their interactions when care planning. Many talked about how the severity of a specific patient’s health or acuity of care needs dictated decisions, feeling the need to account for changes in the patient’s health status, and acknowledged an awareness of interactions between health and the complex patient’s psychosocial context.

*“What's going to kill him the quickest. What, if this went wrong, would lead to the most imminent demise. And that was the prioritization”* (P16).

*“I'll start by asking them how they're doing, if anything has recently changed, if there's any new stressors in their lives that would potentially interfere with their ability to effectively manage their medicine”* (P11).

Besides acuity, most physicians articulated that they prioritized among care needs based on concrete or observable data from complex patients, such as age, finances, mental health, decision-making capacity, labs, or vital signs.

*“I always look to see if they have had recent lab [s] and I look for lab abnormalities, as I'm kind of talking with them, I look at their vital signs and if they've kept a chart for me, that's good, and I look at that. [ … ] I look at all the data that's available”* (P21).

A few physicians, however, felt there was no way to generalize how they made decisions for complex patients.

*“When you asked me what sets the agenda, the answer is it's this deeply matrixed thing”* (P07).

#### Subtheme #2. An underlying PCP style or habit guides decisions

Clinician style impacted care decisions for complex patients. For example, some alluded to wanting to ‘do no harm’ or minimize unnecessary care for vulnerable patients.

*“Trying to determine the most innocuous care plan that takes into consideration his additional comorbidities”* (P24).

*“Polypharmacy is a big problem. […] I was like, wow, is there a way that we can consolidate, or does he really need this?”* (P13).

Some physicians relied on systematic approaches in care planning for complex patients, such as a habit of always arranging follow-up appointments for continuity or pre-visit preparation for efficient interactions. Several described the role of documentation, noting how their care decisions were guided by past records and using the health record to communicate plans to other team members.

*“The first thing I do is I have very good notes, so [the care team] can rely on those. And they're also organized in a way that you can exactly tell what I'm thinking about”* (P21).

#### Subtheme #3. Care planning occurs towards an overarching goal for care

Most physicians felt that maintaining the stability or status-quo of complex patients dictated decisions. Safety was also described as a major goal.

*“First priority is clinical stability. That’s very important, of course. Things that may tip the patient into less clinical stability”* (P17).

Care decisions often prioritized the priorities and concerns of complex patients, frequently focusing on symptoms or function.

*“He has chronic back and leg pain that we discuss pretty much every time I see him, because that's one of his primary concerns”* (P12).

Many discussed deliberately balancing their own and the patient’s goals, and some recognized that family, caregiver, or other healthcare staff goals were influential. Some physicians also described how they tried to obtain goal alignment before feeling able to advance care plans.

*“My usual way of prioritizing is to find out what the most important thing to the patient is, but for him, both the patient and the spouse, and go through their list of priorities and then try to hit my priorities”* (P19).

*“I may have an agenda, but if my agenda doesn't match up with my patient's agenda, then we may not make any progress”* (P19).

### External factors within the environment or context influence care planning decisions

#### Subtheme #4. Patient access to resources impacts care plans

Physicians considered potential limitations on patient access to care when deciding care plans, including resources, matching needs with available options, and travel time burden on patients with multimorbidity.

“*We have no lab, we have no x-ray, we have no nothing. It's just me in a room, that's it*” (P12).

“*When the traffic is good, and it [the drive] could be even up to two hours. So I've been trying to get him imaging outside [the clinic]*” (P15).

Many physicians considered time tradeoffs in prioritizing issues to address at visits. They recognized that the number of issues per encounter was limited by time, and not addressing some needs would mean bringing patients back to clinic more often. Some responded either by opting to extend visits or setting patient expectations. Several also described how more frequent visits weren’t always permissible in the VHA.

“*Let's face it: you get the first two or three [issues] and make sure there's nothing acute going on otherwise and then see him back in a month or two to catch the rest of it*” (P21).

*“I really feel that the more complex patients, we need to be able to see them more often”* (P23).

Some physicians discussed the importance of proactively getting ahead of patient needs, in order to deal with the volume of care needs that might otherwise overwhelm their capacity for patient access to care. One described this process (referencing the Care Assessment Need score, a VHA risk score predicting adverse outcomes of utilization or mortality) [31]:

*“About every quarter, my nurse [ … ], just pulls the CAN score list and anybody with an elevated CAN score she just cold calls him”* (P03).

Finally, some physicians facilitated or organized their care planning decisions that were affected by patient access more reactively, in response to their perception of a patient’s goals as beneficial to health, likelihood of follow-up, or (in)ability to carry out plans.

*“If patients are wishing to, say, continue tobacco use, I'm certainly less likely to try to give them quicker refills on some of their inhaler medications”* (P16).

#### Subtheme #5. Organizational structures provide boundaries

Several physicians felt care decisions were dictated by system features or idiosyncrasies. This included physicians trying to match decisions to the visit format (e.g., in-person or telehealth), and being aware of VHA performance metrics.

*“My plan is to do everything I can at one visit that requires me to examine them”* (P01).

*“Most of the time with this patient, their priorities and my priorities and the quality metrics of the watchdogs are all going to match”* (P23).

A few physicians noted how decisions were affected by the (lack of) availability of information about a patient. Some felt this stemmed from COVID-19 restrictions on bringing patients into the office, while others described how complex patients refused or were unable to make needed disclosures under some circumstances that were affected by organizational processes, such as visit format dictated by clinic protocol.

*“By telephone, with all this going on, especially right now, it's a lot easier for them to say nothing's wrong, and you're just going, ‘Oh, I don't know if I believe you or not’”* (P23).

### Relationship-based factors across individuals affect care-planning decisions

#### Subtheme #6: care planning and delivery for complex patients is collaborative in primary care

Many physicians described working with their medical home teams to decide on or carry out recommendations for their complex patients. In response to high volumes of care needs, they described dividing up tasks among available team members and using their team to extend follow-through and ensure care coordination for ongoing needs. Physicians also described relying on collateral input from their team to care plan.

“*Usually, I would talk to my nurse. She seems to have a lot more ideas. If we can’t get anywhere, we will talk to social work*” (P01).

Specific areas of care for complex patients, however, were felt to fall outside the scope of primary care teams. Mental health was felt by some physicians to be a particularly siloed domain, and others felt that there were some care needs which were the responsibility of specialists.

*“I just found a psychiatrist and invited them to please help me, because I really think this is your job”* (P13).

#### Subtheme #7. Decisions are affected by dynamic patient relationships

Physicians considered their relationship with complex patients and tried to achieve trust and gain treatment buy-in from patients.

*“You have to make your patients feel like you care about them. You should care about them, but you also have to convey that to them, that you do care and their needs and their concerns and what's important to them, it's also important to you”* (P19).

*“When I first met her, it was just laying down the groundwork and essentially I attempted to build a rapport and lay out what the issues are and asked her to consider how we could hopefully come to some communal ground” (P08).*

A few physicians described how they emotionally reacted to some complex patients or had self-imposed boundaries that influenced their decisions.

*“He's a lovely man and it's great to hang out with him and enjoy the experience of meeting with him, but also try and make sure that he really does understand what you are suggesting, and wants to make the kinds of changes you're talking about”* (P07).

Physicians also reflected on how their decision-making may have evolved in response to the influence of prior patient behavior and their experiences within the relationship.

*“I used to automatically refill his medication just to make sure he had them, and then I realized that didn't let me assess what he was actually taking, so I stopped doing that”* (P14).

## Discussion

We conducted a qualitative study of primary care physicians within the VHA to ascertain decision-making factors for care planning for complex patients with multimorbidity. We found that physician decision-making for these patients was governed by internal (individual patient- and physician-specific), external (environmental or contextual), and relationship-based factors. While aspects of our findings could be influential for clinical decision-making more generally, physicians in our study commonly described factors that were more influential due to illness severity, individualized to complex patient circumstances, or conflicting given multiple health needs. We found that care planning for complex patients was matched by equally complex decision-making; physicians made goal-directed decisions incorporating patient health data and psychosocial context, but also responding to their own emotional state and relationship with the patient. The volume of care needs for complex patients was important and cut across several themes, necessitating varied approaches and styles for prioritization and organization. Physicians also described collaborative, team-based decision-making for complex patients within the medical home model, but perceived clear boundaries with practice scope and health system structures.

Few studies have explored the intricacies of physician decision-making during care planning for complex patients with multimorbidity. Prior work describing this process more globally found that decisions are highly varied, with few generalizable conclusions for multimorbidity [21, 32]. In one review, nearly two-thirds of the studies excluded patients with multimorbidity [32]; Other reviews have focused on specific facets of decision-making such as collaborative shared-decision making [33, 34]. Heterogeneity in the decision-making literature may also due to an emphasis on care coordination in multimorbidity care, [35, 36] which requires input from other care team members than physicians. The importance of care coordination was not underestimated by our physician sample, who also referenced care coordination, often within the context of the collaborative medical home model or team-based care. Lack of consensus on decision-making factors in the literature may also arise from the inadequacies of clinical practice guidelines and a dearth of evidence applicable to multimorbidity [37]. To some extent, our findings reflect these elements, both in that our physicians recognized a diversity of factors governing their decisions and in recognizing the importance of other decision-makers to care planning.

Some of our findings echo prior research, adding to our study transferability. As we found, longer encounter times have been described as integral to addressing the multiple care needs of complex patients [21]. In line with prior literature, we found that tension arising from contrasting goals for care are common among different stakeholders, including between more organ or disease-specific specialists and “holistic” PCPs, [21] and between patients prioritizing symptoms and clinicians focusing on long-term prognosis [38, 39]. Clinicians try to resolve these tensions by compromising on conflicting care goals, which our participants also described [21]. This willingness to compromise within the decision-making process is mirrored by similar findings on clinician perspectives on shared decision-making [21, 40]. Finally, patient factors that affect care planning were also acknowledged by our physicians. Limited patient capacity to conduct disease self-management has been described, [41, 42] particularly among those with cognitive impairment, comorbid mental health diagnoses, or low social support [21]. Adherence to care plans, recognition of the importance of polypharmacy and medication reconciliation, and the burden of treatment have all been described as important considerations when care planning for these patients [21, 32, 41, 42].

Our study has important practice implications specific to patients with multimorbidity and/or complexity. First, we found physicians employed clear strategies to effectively make care planning decisions (and conduct related care delivery) for complex patients. High volumes of care needs are a known issue with complex patients [43]. In our sample, physicians responded by conducting proactive outreach to anticipate needs, delegating tasks, using other team members in their medical home (nurses, pharmacists) to conduct follow-up for residual needs, and efficiently gathering collateral information through their team. Accepting that tradeoffs exist in what can be realistically addressed in visits is also described in the literature for complex patients [44]. We found physicians used varied approaches in response, including using longer vs more frequent visits, matching tasks to visit formats (e.g., in-person) and logistics (e.g., travel time), or relying on pre-visit preparation. Beyond these strategies, our study also highlights the intricacies of physician decision-making for complex patients – while illustrating individual factors, the themes collectively represent interacting elements impacting care planning. Individual context, team dynamics, and organizational structures contribute to the “deep matrix” of physician decision-making for complex patients with multimorbidity and reinforce that care planning is itself a complex system [45]. While clinical practice guidelines and organizational recommendations recognizing the individuality and complexity of decision-making for this population may provide some, albeit limited guidance, [46, 47] other assistance could arise from adaptive, shared electronic health records or greater collaborative care infrastructure to advance this style of decision-making for these patients [48, 49].

Our national sample, data collection until thematic saturation was reached, and convergence with prior literature adds to the trustworthiness of our findings. Limitations include that we drew perspectives only from physicians working in an integrated health system (VHA) specifically with veteran patients; our findings may be less generalizable to decision-making for other members of a care team, fee-for-service conditions, or those working with civilian patients. We note our sample may represent only some perspectives within VHA physicians. While not strictly a limitation, we note physicians who participated were highly experienced – with prior average practice experience of over two decades. This greater level of experience could reflect that those with greater interest or perceived expertise in our topic may have been more likely to respond. While our findings may not capture the decision-making of more junior physicians, the experienced physicians in our sample articulated useful decision-making strategies which may help others navigate conflicting or absent clinical guidelines and evidence for this patient population. Our study also took place at the onset of the coronavirus pandemic (COVID-19), which may have impacted experiences of frontline physicians caring for vulnerable patients or influenced which PCPs felt they had time or interest to respond. We have no data on the non-responders to our email recruitment.

## Conclusions

This qualitative study of primary care physicians practicing within patient-centered medical homes in the integrated VHA describes factors affecting care planning decisions for complex patients with multimorbidity. Physicians in our study described internal, external, and relationship-based factors; these insights offer opportunities and practical strategies for health systems and leaders seeking to improve the patient-centeredness and quality of clinical decision-making for complex patients.

## Supplementary Information

**Additional file 1.**

**Additional file 2.**

## Data Availability

The datasets generated and/or analyzed during the current study are not publicly available due to study nature as healthcare operations but are available from the corresponding author on reasonable request.

## Abbreviations

CAN: Care Assessment Need score
COVID: SARs-CoV-2 coronavirus
PCP: Primary care physician
VHA: Veterans Health Administration
