# Supplementary material for: Factors affecting primary care physician decision-making for patients with complex multimorbidity: a qualitative interview study
Source: BMC Prim Care. 2022 Feb 5;23:25. doi: 10.1186/s12875-022-01633-x (PMC8817776; doi:10.1186/s12875-022-01633-x)
Supplement: Supplementary file 1 — Additional file 1. [file 12875_2022_1633_MOESM1_ESM.docx]

| **Standards for Reporting Qualitative Research Checklist** | | |
| --- | --- | --- |
| Topic | Item | Location in manuscript |
| Title | Concise description of the nature and topic of the study Identifying the study as qualitative or indicating the approach (e.g., ethnography, grounded theory) or data collection methods (e.g., interview, focus group) is recommended | Title |
| Abstract | Summary of key elements of the study using the abstract format of the intended publication; typically includes background, purpose, methods, results, and conclusions | Abstract (all). |
| Problem formulation | Description and significance of the problem/phenomenon studied; review of relevant theory and empirical work; problem statement | Background, paragraphs 1-3. |
| Purpose/research question | Purpose of the study and specific objectives or questions | Background, end of paragraph 3. |
| Qualitative approach and research paradigm | Qualitative approach (e.g., ethnography, grounded theory, case study, phenomenology, narrative research) and guiding theory if appropriate; identifying the research paradigm (e.g., postpositivist, constructivist/ interpretivist) is also recommended; rationale | Methods, Study overview and setting (paragraph 1) and Data analysis (paragraph 5). |
| Researcher characteristics and reflexivity | Researchers’ characteristics that may influence the research, including personal attributes, qualifications/experience, relationship with participants, assumptions, and/or presuppositions; potential or actual interaction between researchers’ characteristics and the research questions, approach, methods, results, and/or transferability | Methods, Researcher characteristics and reflexivity (paragraph 2). |
| Context | Setting/site and salient contextual factors; rationale | Background, paragraph 3. Methods, Participants (paragraph 3). |
| Sampling strategy | How and why research participants, documents, or events were selected; criteria for deciding when no further sampling was necessary (e.g., sampling saturation); rationale | Methods, Participants (paragraph 3) and Interviews and data collection (paragraph 4). |
| Ethical Issues | Documentation of approval by an appropriate ethics review board and participant consent, or explanation for lack thereof; other confidentiality and data security issues | Methods, Study overview and setting (paragraph 1). |
| Data collection methods | Types of data collected; details of data collection procedures including (as appropriate) start and stop dates of data collection and analysis, iterative process, triangulation of sources/methods, and modification of procedures in response to evolving study findings; rationale | Methods, Study overview and setting (beginning of paragraph 1) and Interviews and data collection (paragraph 4). |
| Data collection instruments | Description of instruments (e.g., interview guides, questionnaires) and devices (e.g., audio recorders) used for data collection; if/how the instrument(s) changed over the course of the study | Methods, Interviews and data collection (paragraph 4).  Additional File 2 (Interview guide). |
| Units of study | Number and relevant characteristics of participants, documents, or events included in the study; level of participation (could be reported in results) | Methods, Participants (paragraph 3).  Results, paragraph 1 and Table 1. |
| Data processing | Methods for processing data prior to and during analysis, including transcription, data entry, data management and security, verification of data integrity, data coding, and anonymization/deidentification of excerpts | Methods, Interviews and data collection (paragraph 4) and Data analysis (paragraph 5). |
| Data analysis | Process by which inferences, themes, etc., were identified and developed, including the researchers involved in data analysis; usually references a specific paradigm or approach; rationale | Methods, Researcher characteristics and reflexivity (paragraph 2) and Data analysis (paragraph 5). |
| Techniques to enhance trustworthiness | Techniques to enhance trustworthiness and credibility of data analysis (e.g., member checking, audit trail, triangulation); rationale | Methods, Interviews and data collection (paragraph 4).  Discussion, start of paragraph 5. |
| Synthesis, interpretation | Main findings (e.g., interpretations, inferences, and themes); might include development of a theory or model, or integration with prior research or theory | Results (all).  Discussion (paragraphs 2, 3). |
| Links to empiric data | Evidence (e.g., quotes, field notes, text excerpts, photographs) to substantiate analytic findings | Results (all).  Tables 2-4. |
| Integration with prior work, implications, transferability, and contributions | Short summary of main findings; explanation of how findings and conclusions connect to, support, elaborate on, or challenge conclusions of earlier scholarship; discussion of scope of application/ generalizability; identification of unique contribution(s) to scholarship in a discipline or field | Discussion (all). |
| Limitations | Trustworthiness and limitations of findings | Discussion, paragraph 5. |
| Conflict of interest | Potential sources of influence or perceived influence on study conduct and conclusions; how these were managed | Competing interest statement. |
| Funding | Sources of funding and other support; role of funders in data collection, interpretation, and reporting | Funding statement. |
