# Supplementary material for: Factors affecting primary care physician decision-making for patients with complex multimorbidity: a qualitative interview study
Source: BMC Prim Care. 2022 Feb 5;23:25. doi: 10.1186/s12875-022-01633-x (PMC8817776; doi:10.1186/s12875-022-01633-x)
Supplement: Supplementary file 2 — Additional file 2. [file 12875_2022_1633_MOESM2_ESM.docx]

**Interview guide used for physician participants.**

**Interview Guide:**

[Interviewer introduction: I’d like you to think of the last few clinic visits you had recently had with a complex patient with multiple chronic conditions, under whatever circumstances you consider “usual care” for you. (Interviewer clarification, if asked for by participant: Multiple chronic conditions are two or more conditions, including any combination of chronic mental or physical health conditions.)]

1. Tell me about the main items or issues addressed in that visit with the patient.
2. Were there any other care needs or issues you addressed?
3. What was the plan at the end of that visit?
4. Are there any other major care issues that have occurred recently with this patient?
5. Of these issues which you just mentioned for this patient, which of these issues need follow-up?
6. What could happen to the patient if follow-up doesn’t occur, for some reason?
7. If you had limited time to address all of this patient’s issues, how would you prioritize among them?
8. If you were precepting a resident or briefing a colleague who was meeting this patient for the first time, what would you say about this patient to help them approach their care?
9. Have you ever had a goals of care or advanced directive conversation with this patient?
   1. [Optional Probe]: Can you tell me about what you learned about the patient during that conversation?
10. What are this patient’s personal values or priorities for health?
    1. [Optional Probe]: How did you learn that these are the patient’s values and priorities for health?
    2. [Optional Probe]: Has the patient ever directly discussed their values or priorities for health with you?
11. Can you recall a time that a patient’s values or priorities for health changed your care plan? Tell me about that.
12. Is there anything else you’d like to tell me about your care for patients with multiple chronic conditions?
